# The Effects of 5% 5-Aminolevulinic Acid Gel and Red Light (ALAD-PDT) on Human Fibroblasts and Osteoblasts

**DOI:** 10.3390/gels8080491

**Published:** 2022-08-08

**Authors:** Tania Vanessa Pierfelice, Emira D’Amico, Morena Petrini, Assunta Pandolfi, Camillo D’Arcangelo, Natalia Di Pietro, Adriano Piattelli, Giovanna Iezzi

**Affiliations:** 1 Department of Medical, Oral and Biotechnological Sciences, University G. d’Annunzio of Chieti-Pescara, 66100 Chieti, Italy; 2 Center for Advanced Studies and Technology-CAST, University G. d’Annunzio of Chieti-Pescara, 66100 Chieti, Italy; 3 School of Dentistry, Saint Camillus International University of Health and Medical Sciences, Via di Sant’Alessandro 8, 00131 Rome, Italy; 4 Dental School, University of Belgrade, 11000 Belgrade, Serbia; 5 Fondazione Villa Serena per la Ricerca, 65013 Città Sant’Angelo, Italy; 6 Casa di Cura Villa Serena del Dott. L. Petruzzi, 65013 Città Sant’Angelo, Italy

**Keywords:** 5-delta aminolevulinic acid, photodynamic therapy, periodontal tissues, protoporphyrin, reactive oxygen species

## Abstract

This study aimed to evaluate the effects of a new photodynamic protocol (ALAD-PDT), consisting of 5% 5-aminolevulinic acid-gel and 630 nm-LED, already used for antibacterial effects in the treatment of periodontitis, on human gingival fibroblasts (HGF) and primary human osteoblasts (HOB). HGF and HOB were incubated with different ALAD concentrations for 45 min, and subsequently irradiated with 630 nm-LED for 7 min. Firstly, the cytotoxicity at 24 h and proliferation at 48 and 72 h were assessed. Then the intracellular content of the protoporphyrin IX (PpIX) of the ROS and the superoxide dismutase (SOD) activity were investigated at different times. Each result was compared with untreated and unirradiated cells as the control. Viable and metabolic active cells were revealed at any concentrations of ALAD-PDT, but only 100-ALAD-PDT significantly enhanced the proliferation rate. The PpIX fluorescence significantly increased after the addition of 100-ALAD, and decreased after the irradiation. Higher ROS generation was detected at 10 min in HGF, and at 30 min in HOB. The activity of the SOD enzyme augmented at 30 min in both cell types. In conclusion, ALAD-PDT not only showed no cytotoxic effects, but had pro-proliferative effects on HGF and HOB, probably via ROS generation.

## 1. Introduction

The imminent problem of antibiotic and antifungal resistance has prompted research to find alternative or additional treatments [1,2,3]. The use of light at specific wavelengths has shown encouraging results against Gram-negative and -positive bacteria, without any phenomena of resistance documented [4]. In particular, near-infrared light (NIR) has shown the ability to decrease different species of bacteria in vitro, such as *Pseudomonas aeruginosa* and *Enterococcus faecalis* [3,5]. Another advantage of the use of light is the synergic effect of photoinactivation with other antimicrobial substances, such as sodium hypochlorite and chlorhexidine [3,5]; consequently, its use in dentistry and medicine has been proposed. Photoinactivation has been maintained for 1 week following light irradiation [6]. There are many theories about the mechanisms of the action of light-photoinactivation, however, the most accredited hypothesis is the ability of light to stimulate endogenous photosensitizers that trigger a cascade of events that culminate with the production of reactive oxygen species (ROS), that in high concentrations, are cytotoxic [4]. However, the presence of endogenous photosensitizers inside bacteria is not constant, but is influenced by many factors such as culture medium and growth conditions. Additionally, light-photoinactivation is not always consistent, and the effects on bacteria can be completely different, not only among different types of bacteria, but also among different strains, by changing the parameters related to the light irradiations (wavelength, intensity of light, frequency, and time of irradiation) or oxygen concentration during irradiation [7]. To reduce the variability in bacteria-killing due to the presence of endogenous photosensitizers, a novel photodynamic protocol has been introduced: the ALAD-PDT protocol, which consists of the use of a thermosensitive gel containing 5-aminolevulinic acid incubated for 45 min–1 h, and then irradiated with 630 nm red light. During the period of incubation, the aminolevulinic acid is converted into protoporphyrin IX (PpIX), a photosensitive molecule [8]. As known, bacteria and neoplastic cells accumulate a high quantity of intracellular PpIX due to their high turnover and lack of ferrochelatase, the key enzymes of heme biosynthesis [9].

Consequently, the 630 nm light irradiation of cells with an increased accumulation of PpIX promotes a highly selective production of ROS, and decreased levels of mitochondrial membrane potential, which induces cell death by both apoptosis and necrosis without damaging the surrounding healthy cells [9]. The ability of ALAD-PDT to inactivate both Gram-positive and -negative bacteria has been shown in different in vitro studies [10,11,12]. ALAD-PDT is effective for biofilm removal from different titanium surfaces, showing encouraging results against peri-implantitis and chronic wounds [11,13]. Incubation with ALAD followed by 630 nm light irradiation was also shown to be effective against *Candida albicans*, in vitro [14]. Lauritano et al. showed that periodontal sites treated with scaling root planing (SRP) and ALAD-PDT showed a lower bacterial load 15 days after treatment, than SRP alone [15]. These effects were translated into clinical parameters at 6 months; indeed, the group treated with SRP and ALAD-PDT showed significantly lower values of periodontal pocket depth (PPD) and clinical attachment loss (CAL), with respect to SRP alone [15]. Clinically, the healing of periodontal pockets is not only the consequence of bacterial removal but also the result of an ordered, integrated, and dynamic process that involves the activation of different cell components, cytokines and growth factors [16,17]. Periodontal regeneration requires restoration of alveolar bone height, formation of new extrinsic-fiber cementum attached to root surfaces, synthesis of Sharpey’s fibers that insert into cementum, and re-establishment of a soft-tissue seal at the gingival interface [17]. These clinical results are indeed the consequence of the additional antibacterial activity promoted by ALAD-PDT. However, not much information is available about the effects of this specific protocol on periodontal tissues, particularly on osteoblasts and fibroblasts. Firstly, this protocol should not be toxic for these cells. Still, it would be desirable if ALAD-PDT could promote cellular proliferation and bone deposition to boost the process of healing.

Thus, the aim of this study was to evaluate the effects of ALAD-PDT on human gingival fibroblasts (HGF) and oral osteoblasts (HOB), in order to gain more information about the mechanisms of the healing of periodontal tissues treated with this novel protocol. Firstly, the viability and the morphology at 24 h, and the proliferative activity of cells at 48 and 72 h after treatment, were evaluated with different concentrations of ALAD followed by LED irradiation. Given that the photodynamic mechanism leads to the accumulation of PpIX and elevated ROS production in cells such as microbes and neoplastic cells, the PpIX intracellular content and ROS levels were measured at different times. The activity of an antioxidative enzyme such as superoxide dismutase (SOD) was also investigated.

## 2. Results and Discussion

Previous results of in vitro and in vivo studies concerning the ALAD-PDT protocol applied to treat oral infections by killing microbes [10,11,12,15] drove us to test the same protocol to investigate whether ALAD-PDT may have some effects on cell populations of the oral cavity such as gingival fibroblasts and oral osteoblasts derived from human biopsies.

In this study, the impact of ALAD, a gel containing 5% of 5-aminolevulinic acid, was examined with and without red light irradiation (PDT). Of specific interest was the determination of the poorly investigated cellular response of fibroblasts and osteoblasts to ALAD-PDT that is applied topically as an antimicrobial [15,18], considering as a goal the lack of cytotoxicity of the surrounding tissue.

### 2.1. Cell Survival and Morphology after ALAD-PDT

**Figure 1:**
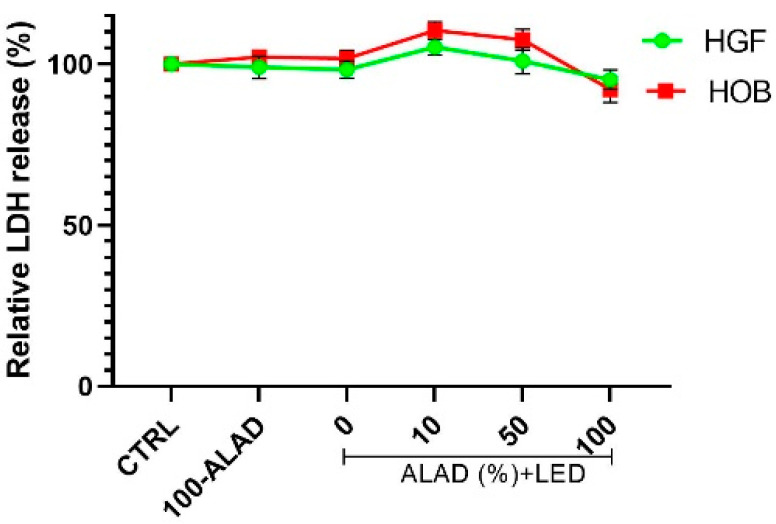
Cytotoxicity assay. HGF and HOB were subjected to ALAD-PDT treatment, and LDH release was measured at 24 h. The data are expressed as percentages with respect to the control (CTRL).

Firstly, the cytotoxicity of different concentrations of ALAD in combination with light was assessed at 24 h. The cytotoxicity of 100-ALAD alone, and light without ALAD, was also evaluated. In this study, both cell types showed a similar response to ALAD-PDT that resulted as not cytotoxic for cells that released a small quantity of LDH after incubating with different concentrations of ALAD and then being exposed to irradiation (Figure 1). Cultured HOB subjected to 100-ALAD-PDT exhibited higher viability than control cells and HGF. Neither irradiation in the absence of ALAD loading, nor incubation with ALAD alone in the absence of irradiation, revealed cellular toxicity.

**Figure 2:**
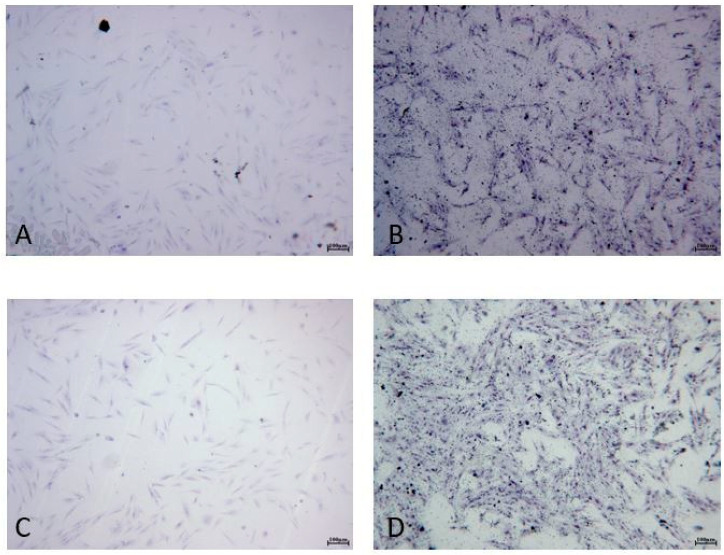
Cell morphology. HGF were subjected to ALAD-PDT protocol and stained after 24 h with toluidine blue. (**A**) CTRL, (**B**) 0-ALAD-PDT, (**C**) 100-ALAD, (**D**) 100-ALAD-PDT.

**Figure 3:**
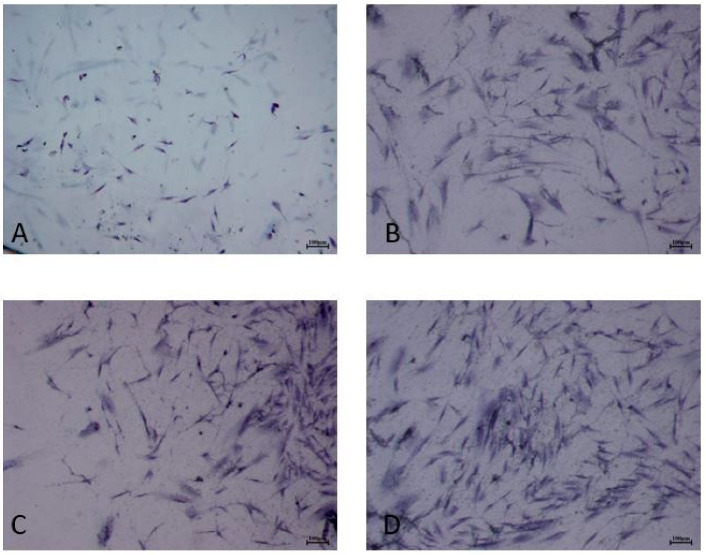
Cell staining. HOB were subjected to ALAD-PDT protocol and stained after 24 h with toluidine blue. (**A**) CTRL, (**B**) 0-ALAD-PDT, (**C**) 100-ALAD, (**D**) 100-ALAD-PDT.

For this reason, the morphology of cells was investigated only for 100-ALAD with and without irradiation at 24 h. The staining with blue toluidine showed that HGF and HOB exhibited similar morphological behavior, confirming the results of the LDH assay (Figure 2 and Figure 3). Indeed, HGF (Figure 2) and HOB (Figure 3) appeared spindle-shaped and well spread after ALAD-PDT treatment.

### 2.2. Cell Proliferation after ALAD-PDT

**Figure 4:**
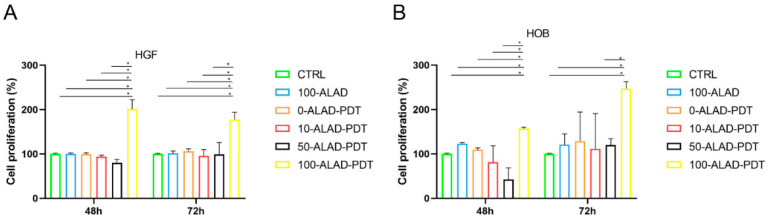
Cell proliferation assay. HGF (**A**) and HOB (**B**)were treated with different concentrations of ALAD gel and exposed to 630 nm LED. Cell growth was measured after 48 h and 72 h. Data are expressed in percentages with respect to control (CTRL). Data are presented as mean ± SD (error bars) of three independent experiments. The proliferation rate was significantly higher with the complete treatment 100-ALAD-PDT at 48 h for HGF, and 72 h for HOB. (* *p* < 0.0001).

Based on the results of the LDH, both cell types were incubated with increasing doses of ALAD for 45 min, and after that, exposed to light to assess their proliferative activity using the MTS assay at 48 h and 72 h. The number of viable and metabolically active cells were significantly higher when ALAD-PDT was applied at a concentration of 100% (Figure 4). The 100-ALAD, in combination with the LED, was more effective in promoting the proliferation of HGF at 48 h and of HOB at 72 h. This response time was in line with the biology of the cell types [19,20,21]. Fibroblasts were not affected by ALAD-PDT, with ALAD concentrations below 100% compared with untreated control cells. Neither irradiation in the absence of ALAD incubation (0-ALAD-PDT), nor incubation with ALAD alone in the absence of light (100-ALAD) promoted HGF activity in respect of control cells (Figure 4A). In contrast, viable and proliferative osteoblasts were also observed in the presence of 100-ALAD without subsequent LED irradiation. The proliferation rate of osteoblasts resulted as significantly higher with 100-ALAD-PDT mainly at 72 h (Figure 4B). The literature showed contrasting results concerning the effects of the 5-aminolevulinic acid on normal cells [22,23,24]. Egli R.J. in 2007, and Bastian J.D. in 2008, showed cytotoxicity in different cell types after the application of the same photodynamic protocol consisting of incubation with 5-aminolevulinic acid for 4 h and subsequent irradiation with laser devices at high light doses [22,23]. In contrast, Kushibiki and co-workers observed photochemical promotion of the murine osteoblast’s differentiation by applying low-light irradiation [24].

The approach in the present study is different in terms of gel incubation and irradiation times, 45 and 7 min, respectively, also considering that one of the aims of clinicians is to reduce both the working time and the patient’s compliance.

### 2.3. ALAD Induces Accumulation of PpIX

5-aminolevulinic acid induces the production of the endogenous photosensitizer PpIX, which is a precursor in heme biosynthesis [25]. PpIX accumulation is selective in pathological tissues without causing changes to the surrounding healthy tissues [25,26].

**Figure 5:**
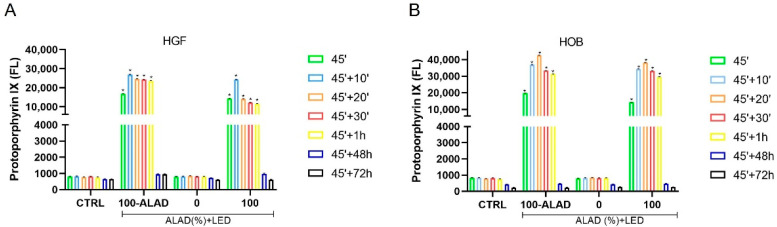
Levels of intracellular PpIX. Fluorescence of PpIX was measured (λex = 405 nm; λem = 608 nm) immediately after the end of ALAD-PDT (45′) and after 10 min (45′ + 10′), 20 min (45′ + 20′), 30 min (45′ + 30′), 1 h (45′ + 1 h), 48 h (45′ + 48 h) and 72 h (45′ + 72 h). Data are presented as mean ± SD of three independent experiments. The statistical analysis was performed by ANOVA test comparing each value to its control (* *p* < 0.0001). A peak of PpIX fluorescence was detected at 10′ for HGF (**A**) and at 20′ for HOB (**B**).

The results of the PpIX fluorescence measured in fibroblasts and osteoblasts, indicated an increment of PpIX that occurred within 1 h (1 h) after the incubation time (45 min) with ALAD (Figure 5). However, the fluorescence peaks were observed at different times based on the cell types. PpIX emitted the highest fluorescence at 10 min for HGF (Figure 5A) and 20 min for HOB (Figure 5B). A previous study evidenced how PpIX differently accumulated in different cell types; the two factors that determine the intracellular levels of PpIX are the rate of cellular uptake of 5-ALA and the intracellular synthesis of PpIX from 5-ALA [27]. The subsequent exposure to LED light provoked the decrease in the fluorescence of PpIX within 1 h following treatment, and returned to baseline levels such as in the control group at 48 and 72 h. This was in agreement with the literature, where the immediate effect of photoactivation of the photosensitizer leads to its photobleaching [28]. The level of PpIX fluorescence was in line with the promotion of proliferation, that were both higher in osteoblasts than in fibroblasts, indicating a positive effect of PpIX on the metabolic activity of cells.

### 2.4. Intracellular ROS Levels after ALAD-PDT

**Figure 6:**
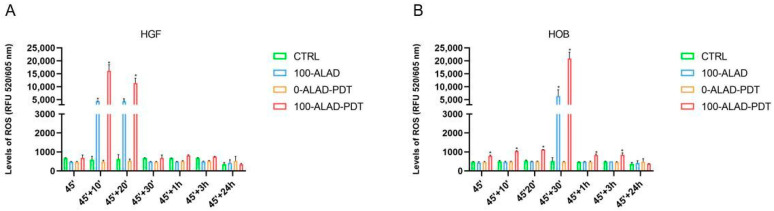
Levels of ROS. The quantification of ROS was measured after the ALAD-PDT protocol at the end of the treatment (45′), at 10 min (45′ + 10′), at 20 min (45′ + 20′), at 30 min (45′ + 30′), at 1 h (45′ + 1 h), at 3 h (45′ + 3 h), and at 24 h (45′ + 24 h). Data are reported as mean ± SD of three independent experiments and expressed in the relative fluorescent unit (RFU), measured at λex/em 520/605 nm. The largest increment was observed after 10′ for HGF (**A**) and 30′ for HOB (**B**). The statistical analysis was performed by ANOVA test comparing each value to its control (* *p* < 0.0001).

During the photodynamic process, ROS are generated in cells, once the photosensitizer molecule, such as PpIX, is photoactivated by light. Thus, the damage caused by ROS is at the basis of the photokilling of cancer and bacteria cells. This aspect drove us to investigate if a high level of ROS were also generated in healthy cells after the ALAD-PDT protocol, given that the PpIX result increased. The fluorescence of PpIX was elevated within 1 h after the application of 100-ALAD-PDT and dropped at 48 h; therefore, in this study, ROS were measured until 24 h after the photodynamic protocol. The results showed that intracellular ROS were significantly enhanced by 100-ALAD compared with the control cells, mainly after LED irradiation (100-LAD-PDT). However, there were some differences accordingly to the cell type. In HGF the enhanced ROS were detected after 10 and 20 min following the ALAD-PDT treatment, with the highest peak at 10 min. ALAD alone (100-ALAD) also increased the production of ROS at 10 and 20 min (Figure 6A), whereas in HOB, ALAD-PDT significantly stimulated the ROS after 30 min in respect to basal concentrations of ROS in control cells (Figure 6B). The ROS levels in both cells showed a similar result to the control group by 1 h after the photodynamic protocol, and also remained at the basal level at 24 h. Studies have shown that high levels of ROS can cause cell death, but ROS are also important mediators of intracellular signaling [29,30,31]. Although the detailed mechanism has yet to be revealed, Kushibiki et al. showed that PDT promotes murine osteoblasts differentiation via AP-1 that is upregulated by high ROS production [24]. Here, the osteoblasts showed higher production of ROS in respect to the fibroblasts.

### 2.5. SOD Activity after ALAD-PDT

**Figure 7:**
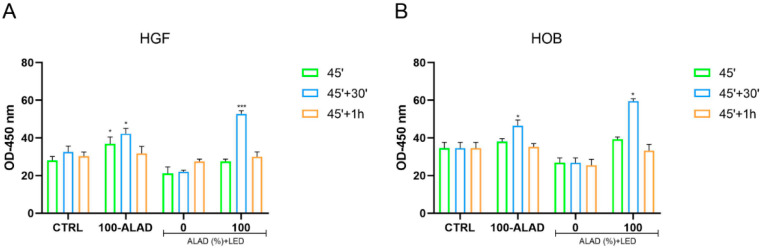
SOD activity. Levels of SOD were detected after the application of ALAD-PDT protocol (45′) and after 30′ and 1 h from its end. Data are expressed as mean ± SD and measured as optical density (OD). A higher SOD activity was observed in ALAD-PDT treated cells after 30′ for both cell lines. The statistical analysis was performed by ANOVA test comparing each value to its control (* *p* < 0.05; *** *p* < 0.0001).

To detoxify unregulated ROS, cells modify antioxidant enzymes such as superoxide dismutase enzyme (SOD). SOD converts superoxide to hydrogen peroxide, which is then removed by glutathione peroxidase or catalase. Thus, SOD prevents the formation of highly aggressive ROS, such as peroxynitrite or the hydroxyl radical [32]. ROS were generated in a time range between 10 min and 30 min after the application of 100-ALAD-PDT, and decreased at 1 h; therefore, the activity of the SOD enzyme was observed until 1 h after the photodynamic protocol. In this study, both HGF and HOB showed a similar trend to the activity of this antioxidative enzyme (Figure 7), even if the levels of SOD activity were lower in HGF (Figure 7A) than HOB (Figure 7B). A higher SOD activity was observed in ALAD-PDT-treated cells, compared with the control, with the highest peak at 30 min after the treatment. ALAD alone (100-ALAD) slightly enhanced SOD activity. In contrast, SOD activity was higher in control cells compared with cells experiencing LED light alone (0-ALAD-PDT). There was not a significant difference between the patterns of ROS and SOD. All aerobic organisms have multiple SOD proteins targeted to different cellular and subcellular locations, reflecting the rate of diffusion and multiple sources of their substrate superoxide [32]. In addition, the SOD-catalyzed dismutation reaction is extremely efficient, occurring at the almost diffusion-limited rate of ∼2 × 10^9^ M^−1^·s^−1^, which is ∼104 times the rate constant for spontaneous dismutation [33]. In this study, the highest peak of ROS and SOD both occurred at the same time (30 min). Moreover, superoxide is relatively short lived [34]; thus, SOD also acts rapidly, and also has a short life. In our study, SOD levels returned to baseline levels, similar to the control group, within 1 h following the photodynamic protocol. These results may indicate that ALAD-PDT application plays a complementary role in ROS production and in the maintenance of SOD activity to counteract ROS.

Altogether, these results might suggest that although ALAD-PDT is used primarily as an antibacterial and antifungal therapy, it seems to have pro-proliferative effects on HGF and HOB via PpIX increment, which lead to ROS generation. It has been reported that ROS trigger cell proliferation and regulate cell differentiation [35,36,37]. In this study, osteoblasts seemed to be more sensitive to ALAD-PDT than fibroblasts. Several authors have reported contrasting results of the application of 5-aminolevulinic acid on human and animal cells [23,24,27]. The novelty of the ALAD-PDT protocol consists of a new formulation of 5% 5-aminolevulinic acid gel, patented (PCT/IB2018/060368, 19 December 2018), that permits a reduction of the time of gel incubation (45–60 min) and reduces the light dose (23 J/cm^2^). The peculiar characteristics of ALAD gel are determined by the presence of a poloxamer mixture in the formulation that facilitates the 5-aminolevulinic acid to rapidly access into target cells. Current research is looking for new approaches that can be bactericidal but also have advantages over traditional antibiotic therapy, and aPDT has been researched as an alternative and promising method for eradicating oral pathogenic bacteria; recent studies have focused on the effects of different photosensitizers [38,39]. Compared with other photosensitizers, 5-aminolevulinic acid as the active ingredient of ALAD gel showed two main advantages: it acted as a pro-drug, inducing PpIX production, which is an endogenous molecule. Studies have shown the effectiveness of this gel in combination with LED against Gram-negative and -positive bacteria, without any phenomena of resistance documented, and also against oral biofilms [10,14,15]. In addition, this preliminary study shed light on the beneficial effects of ALAD gel on cell populations of the oral cavity that potentially enter into contact with gel when it is applied as a PDT.

Furthermore, several authors have reported the use of gels for wound healing, bone regeneration, and the treatment of inflammatory diseases [40,41,42,43,44]. In this study, the enhanced metabolic activity induced by the ALAD-PDT protocol may suggest a new mechanism for fibroblast and osteoblast proliferation.

## 3. Conclusions

In conclusion, the ALAD-PDT protocol, consisting of a new formulated gel based on 5% 5-aminolevulinic acid, that has already demonstrated antibacterial effects, was also shown in this study to promote the metabolic activity of gingival fibroblasts and oral osteoblasts, probably via ROS bursting. Although further investigations are needed, the results of this study should expand the utility of the ALAD-PDT protocol in basic research and in clinical applications. Indeed, this preliminary study shed light on the beneficial effects of ALAD-PDT on cell populations of the oral cavity that potentially enter into contact with gel when it is applied as an antimicrobial method. It is also worth noting that the new formulation based on poloxamer mixture allowed the gel to easily adhere to oral mucosae and avoided 5-aminolevulinic acid being washed out by saliva. Finally, the considerable strength of the ALAD-PDT protocol was the shortness of incubation and irradiation times (45 min and 7 min) with respect to photodynamic therapy based on 5-ala generally proposed in the literature.

## 4. Materials and Methods

### 4.1. Experimental Design

The experiments were performed using primary cells harvested from human biopsies. Cells were incubated for 45 min with a gel containing 5% of 5-aminolevilinic (ALAD), commercialized as Aladent by ALPHA Strumenti s.r.l. (Melzo, MI, Italy) in a serum-free medium. An incubation time of 45 min was chosen based on previous works [10,14,15]. Then, the cells were exposed to red LED light (630 nm) with an intensity of 380 mW/cm^2^ (ALPHA Strumenti s.r.l.) for 7 min with a light dose of 23 J/cm^2^. Subsequently, the cells were cultured in a medium containing 10% fetal bovine serum (FBS, Corning, NY, USA). The effects of ALAD-PDT on LDH release, proliferation, PpIX cell accumulation, ROS levels and SOD activity were assessed at different time points. All experiments were performed in triplicate, employing different cell strains every time.

### 4.2. Cell Culture and Isolation

Human gingival fibroblasts (HGF) and human oral osteoblasts (HOB) were obtained from 12 human biopsies of volunteers managed by the dental clinic of the G. D’Annunzio University following a protocol approved by the Ethics Committee of the University of Chieti-Pescara (reference numbers: N. 1968-24.07.2020; BONEISTO N. 22-10.07.2021). Gingival fibroblasts and oral osteoblasts were extracted and cultured as described by Petrini, M. et al., 2021, and Pierfelice, T.V. et al., 2022, respectively [45,46].

### 4.3. Treatments

Cells were seeded, and after 24 h of culture were incubated for 45 min with increasing concentrations (10%, 50%, 100%) (v/v) of ALAD in serum-free medium at 37 °C and 5% CO_2_, and five test groups were distinguished:

-(100-ALAD) were cells treated with ALAD gel at 100% without LED irradiation; 

-(0-ALAD-PDT) were cells exposed to 7 min of LED light irradiation alone, without ALAD addition; 

-(10-ALAD-PDT) were cells treated with ALAD gel (10%) and exposed to LED for 7 min; 

-(50-ALAD-PDT) were cells treated with ALAD gel (50%) and exposed to LED for 7 min; 

-(100-ALAD-PDT) were cells treated with ALAD gel (100%) and exposed to LED for 7 min. Untreated (without ALAD) and unexposed (without LED irradiation) cells were considered as the control group (CTRL).

### 4.4. Lactate Dehydrogenase (LDH) Release Assay

Cytotoxicity was quantified by detecting the activity of LDH released into the cell culture supernatants of 1 × 10^3^ cells/well after ALAD-PDT treatments at 24 h. LDH release was determined by a cytotoxicity detection kit LDH (Roche, Basilea, Switzerland) according to the manufacturer’s protocol. The absorbance was read at 490 nm using a microplate reader (Synergy H1 Hybrid BioTek Instruments, Winooski, VT, USA). LDH release was calculated as a percentage with respect to the control (CTRL).

### 4.5. Toluidine Blue Staining

An amount of 2 × 10^4^ cells/well were seeded and subjected to the ALAD-PDT protocol. After 24 h, adherent cells were fixed with 70% cold ethanol and stained with 1% toluidine blue and 1% borax (Sigma Al-drich, St. Louis, MO, USA). Cells were then observed by microscopy connected with a camera at 40x (Leica, Wild Heer-brugg, Wetzlar, Germany).

### 4.6. Cell Proliferation Assay

Cell proliferation was determined by CellTiter 96 assay (MTS, Promega, Madison, WI, USA). An amount of 1 × 10^4^ cells/well were seeded into 96-well plates for 24 h and treated with ALAD-PDT as reported above. After 48 h and 72 h, MTS solution (10 μL) was added to each well, followed by incubation at 37 °C for 2 h. The absorbance was determined at 490 nm using a microplate reader (Synergy H1 Hybrid BioTek Instruments) and the cell proliferation rate was calculated as a percentage with respect to the control.

### 4.7. PpIX Fluorescence

PpIX intracellular content was determined immediately after the treatment (45′) and at different time points after the treatment, namely, 10 min (45′ + 10′), 20 min (45′ + 20′), 30 min (45′ + 30′) and 1 h (45′ + 1 h). An amount of 6 × 10^3^ cells/well human fibroblasts and osteoblasts were plated in 96-well plates and subjected to the ALAD-PDT protocol according to the paragraph of treatment. Then, cells were treated with a solution of 0.5 M perchloric acid (HClO_4_) in 50% methanol [47] and PpIX fluorescence was measured using a microplate spectrofluorometer (Synergy H1 Hybrid BioTek Instruments) at λex/em 405/608 nm.

### 4.8. Reactive Oxygen Species (ROS) Levels

ROS levels were determined by Cellular Reactive Oxygen Species Detection Assay Kit (Abcam, Cat No. ab186027, Cambridge, UK), according to the manufacturer’s protocol. An amount of 1 × 10^4^ cells/well were seeded in 96-well plates for 24 h of culture. After ALAD-PDT, ROS levels were measured immediately after treatment (45′) and at different time points after the treatment, namely, 10 min (45′ + 10′), 20 min (45′ + 20′), 30 min (45′ + 30′), 1 h (45′ + 1 h), 3 h (45′ + 3 h) and 24 h (45′ + 24 h). A volume of 100 µL/well of ROS Red working solution was added into each well. The plate was incubated at 37 °C for 1 h and the fluorescence at λex/em 520/605 nm was measured by a microplate spectrofluorometer (Synergy H1 Hybrid BioTek Instruments).

### 4.9. Superoxide Dismutase (SOD) Assay

An amount of 2 × 10^6^ cells/well were cultured in 96-well plates, and after 24 h were treated with ALAD-PDT. SOD activity was determined immediately after the treatment (45′) and at different time points after the treatment, namely, 30 min (45′ + 30′) and 1 h (45′ + 1 h), using a SOD assay kit (Abcam, Cat No. ab65354) according to the manufacturer’s instruction. After the preparation of samples, 20 µL of enzyme working solution was added to each well. After incubation of 20 min at 37 °C, the absorbance was measured at 450 nm using a microplate reader (Synergy H1 Hybrid BioTek Instruments). SOD activity was calculated as a percentage of inhibition rate.

### 4.10. Statistical Analysis

All experiments were performed in triplicate and repeated three times. The data are reported as means ± standard deviation (SD). Statistical analyses were performed using the GraphPad Prism8 (GraphPad Software, San Diego, CA, USA). Differences between groups were assessed with one-way analysis of variance (ANOVA). A *p*-value ≤ 0.05 was considered as significant.

## Data Availability

Not applicable.
